# Passage relevance models for genomics search

**DOI:** 10.1186/1471-2105-10-S3-S3

**Published:** 2009-03-19

**Authors:** Jay Urbain, Ophir Frieder, Nazli Goharian

**Affiliations:** 1 Electrical Engineering and Computer Science Department, Milwaukee School of Engineering, Milwaukee, WI, USA; 2 Information Retrieval Lab, Illinois Institute of Technology, Chicago, IL, USA

## Abstract

We present a passage relevance model for integrating syntactic and semantic evidence of biomedical concepts and topics using a probabilistic graphical model. Component models of topics, concepts, terms, and document are represented as potential functions within a Markov Random Field. The probability of a passage being relevant to a biologist's information need is represented as the joint distribution across all potential functions. Relevance model feedback of top ranked passages is used to improve distributional estimates of query concepts and topics in context, and a dimensional indexing strategy is used for efficient aggregation of concept and term statistics. By integrating multiple sources of evidence including dependencies between topics, concepts, and terms, we seek to improve genomics literature passage retrieval precision. Using this model, we are able to demonstrate statistically significant improvements in retrieval precision using a large genomics literature corpus.

## Background

Traditional retrieval functions, including state-of-the-art *probabilistic *and *language models *are typically based on a *bag of words *assumption where text is represented as unordered sets of terms, and any notion of concept identification, term ordering, or proximity is lost. Capturing a greater number of distinct query concepts within the context of a passage of text, however, is more likely to be relevant than a document containing fewer concepts dominated by higher *IDF *or *term frequency *scores. Without modeling contextual dependencies between terms, traditional models are not suitable for disambiguating terms and identifying relevant text without explicit term matching. These issues are particularly relevant when attempting to retrieve passages of text from biological literature where the significant use of ambiguous terms, acronyms, and term variants make identification of biological concepts especially challenging. We use *concepts *here to refer to the meanings, or definitions of natural language *terms*, where *concepts *can be represented by one or more *terms*, and terms can consist of one or more *words*.

Use of external knowledge sources coupled with query expansion techniques have been popular methods for identifying concept term variants. For example, bovine *spongiform encephalopathy*, *BSE*, and *Mad Cow Disease *all refer to the same biological concept. Use of external knowledge sources, however, can be problematic. An acronym like *IP *could represent *immunoprecipitant *or *ischemic precondition*. In this case we can only disambiguate *IP *if we have sufficient context to understand that one of the topics covered in the document involves *immuno precipitation *versus *cardiology*. These techniques also provide no relevance weight to passages, which are contextually similar but lack explicit matching of key terms. For example, acronyms for *immunoglobulin G *can be abbreviated as *IGG, Ig G*, or *IgG*. Since all capitals are frequently used in knowledge sources such as the UMLS, a query augmented with *IGG *would fail to match more general and alternative forms such as *IG *or *IgM *using standard *gene *and *protein *name normalization techniques [1].

Dealing with general concepts like *gene*, *protein*, or *disease*, can be especially troublesome. First, knowledge sources can generate an intractable number of query expansion terms. Second, general concepts take on a more specific meaning when coupled with contextual information. For example, a general term like *protein *when used within the context of the topic *chronic wasting disease *is likely to refer to a *prion protein*. A term like *progression *when used within the topic *neoplasm *is likely referring to *tumor progression*.

To address these issues, we present a passage retrieval model for capturing semantics through the notion of *topic *and *concept relevance *by learning the latent relationships between terms and concepts in *relevant *passages. First we present our passage relevance model, followed by the model's component topic, concept, term, and document models. Next, we review our dimensional indexing and query processing strategies. Finally, we present our results and discussion of prior work.

## Methods

### Passage relevance model

Our passage relevance model is based on the framework of an undirected probabilistic graphical model (Markov Random Field). A *graphical model *is a graph that models the joint probability distribution over a set of random variables. Each node in the graph is a random variable and missing edges between nodes represent conditional independencies. By modeling conditional independence assumptions, the full joint distribution can be factorized into a typically much more manageable product of conditional distributions. Unlike directed graphical models, Markov Random Fields (MRF) are unable to represent induced dependencies (causality) between random variables. This can allow more modeling flexibility, including the ability to model cyclic dependencies and more freedom in defining component models expressed as potential functions over cliques of random variables.

We define our model based on the belief that effective retrieval of relevant passages requires a model for integrating syntactic and semantic evidence from multiple levels of document context. Context is captured at the document level using term statistics, at the passage level through query topic modeling, and at the concept level by identifying concept terms and the terms they co-occur with within the context of a sentence. We posit that the most relevant passages contain the maximum number of distinct query *concepts *and *terms *within the minimum spanning lexical distance. We define passages as one or more contiguous sentences identified from the minimum spanning distance of query concepts.

**Figure 1:**
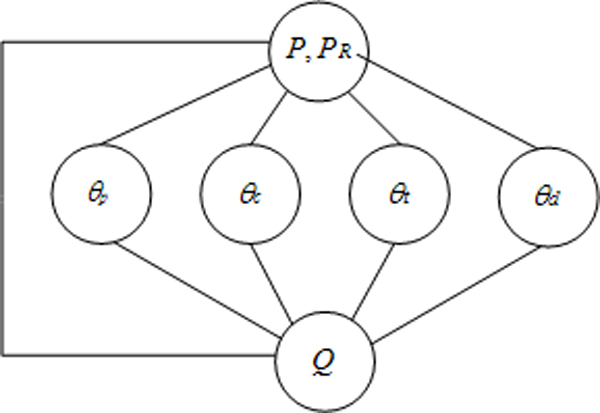
**Passage Relevance Model**.

As shown in Figure 1, our proposed *passage relevance model *represents the joint probability of query *Q *and passage *P *as an undirected graphical model. Edges in the graph define conditional independence assumptions between the component models. The joint distribution across potential functions in the graph represents the probability of a passage being relevant to a biologist's information need. Models for *topic, concepts, terms*, and document *θ*_*p*_, *θ*_*c*_, *θ*_*t*_, *θ*_*d *_respectively, are represented as random variables in the graph. Random variable *P *represents the distribution of features present in the passage without relevance estimates, and *P*_*R *_represents a refinement to this distribution using a *relevant *set of passages. We use the top ranked passages retrieved from the model without using the relevant set as an estimate for *P*_*R*_.

Based on conditional independence assumptions, the model is factorized into a set of maximal cliques (fully connected subgraphs). The joint probability distribution is written as a product of potential functions *ψ*(*c*) over the maximal cliques in the graph (1).

(1) $$p(Q,P)=\frac{1}{Z}\prod_{c\in C(G)}\psi (c)$$

Potential functions in the passage retrieval model are defined for the *topic, concept, term*, and *document *cliques:

(2) *ψ*(*Q*, *P*, *P*_*R*_, *θ*_*p*_) *ψ*(*Q*, *P*, *P*_*R*_, *θ*_*c*_) *ψ*(*Q*, *P*, *P*_*R*_, *θ*_*t*_) *ψ*(*Q*, *P*, *θ*_*d*_)

The joint probability of a query *Q *and passage *P *across all potential functions results in the following:

(3) $$p(Q,P)\propto \sum_{c\in C(G)}\log(\psi (c))$$

Since all potential functions are restricted to being strictly positive, it is customary to express them as exponentials, where *f(c) *is a real valued feature function over clique values and *λ*_*c *_is the weight given to the feature. As we are interested in the *relative *likelihood of each ranked passage within a potential function being *relevant*, and to eliminate parameter tuning, we set all feature function weighting constants *λ*_*c *_to 1, and normalize each function to between *0 and 1 *(4).

(4) *ψ*(*c*) = exp(*λ*_*c*_*f*(*c*)), log(*ψ*(*c*)) = *λ*_*c*_*f*(*c*) = *f*_*norm*_(*c*)

It is important to appreciate that *no *tuning parameters have been introduced to adjust the weight contributed by each potential function. Instead we rely on the relative likelihood of relevance expressed by each potential function. This notion follows from Robertson's probability ranking principle (PRP) [2]. Next, we present the passage model's component topic, concept, term, and document models.

### Topic relevance model

Topics are fundamentally based on the distribution of terms within and across documents [3]. In prior efforts [4], we generated a corpus wide topic model using an unsupervised Markov Chain Monte Carlo sampling procedure. We evaluated the topic model in isolation and as a component within a probabilistic graphical retrieval model. The overall results from the retrieval model were excellent, however the topic model component did not significantly improve results, was difficult to parameterize, and was computationally expensive. These results were consistent with Azzopardi, Girolami, and van Rijsbergen [5], and Wei and Croft [6]. The technique effectively identified related words for automatically generated topics; however these topics were not necessarily relevant to the topic of a user query.

Our objective in modeling *topic relevance *is to directly address the issue of learning a topic model that is *relevant *to the latent structure, or *topic*, of a user query by capturing the probability of each term over all other terms in a relevant set of passages.

Given a relevant set of passages, we can directly estimate the topic distribution of the user query over words. From this distribution we can infer the relevance of any given word by its probability of co-occurrence with all other words in the relevant set [7]. Finally, we can construct a model estimating the relevance of any given passage to a *query *topic from the underlying probability of relevance from its component terms. We start with a Bayes model to estimate the probability of a word *w*_*i *_given the relevant set of passages *R *(5).

(5) $$p(w_{i}|R)=\frac{p(R|w_{i})p(w_{i})}{p(R)}$$

The prior of word *w*_*i *_belonging to the relevant class is estimated by the odds of *w*_*i *_appearing within a relevant passage (6).

(6) $$p(w_{i})=\frac{p(w|R)}{p(w|\neg R)}=\frac{C_{w_{i}\in S_{R}}^{\left|S_{R}\right|}+\beta }{C_{w_{i}\in S,w_{i}\notin S_{R}}^{\left|S\right|}+|S_{R}|\cdot \beta }$$

|*S*_*R*_| is the size of the set of relevant passages.

$C_{w_{i}\in S_{R}}^{\left|S_{R}\right|}$ is the count of relevant passages containing *w*_*i*_.

$C_{w_{i}\in S,w_{i}\notin S_{R}}^{\left|S\right|}$ is the count of paragraphs in the collection containing *w*_*i *_sans the relevant passage count. This serves as a proxy for the count of non-relevant passages.

*β *is a smoothing parameter set to zero, since only terms occurring in at least two relevant passages are considered.

To capture the latent relationships between co-occurring terms in the relevant set, we define the likelihood of relevance *p*(*R*|*w*_*i*_) for *w*_*i *_recursively over all terms co-occurring with *w*_*i *_within a sentence of the relevant set (7).

(7) $$p(R|w_{i})\approx \alpha (\frac{1}{Z}\sum_{j\neq i}^{K}p(R|w_{k}))+(1-\alpha )\cdot p(w_{i})$$

*Z *is normalization constant such that the sum of the probabilities of all distinct relevant terms equals one. Finally, we define the probability of a query *q *for a given passage *m*_*j *_using the sum rule from the underlying *topic relevance *model *θ*_*R*_.

(8) $$p(q|m_{j})=p(m_{j}|\theta_{R})=\sum_{i}^{W}p(R|w_{i})$$

As a proxy for the relevant set of passages, we sample terms from the top *30 *ranked passages containing at least one resolved *concept *using the *passage retrieval model *(Figure 1) *without *using the discrete random variable representing *topic relevance *which we seek here to create. The full model is then evaluated on the top *500 *retrieved passages for final ranking.

In Table 1 we show the top 30 *topic relevance *terms learned for queries 200, 201, and 202 of the 2007 TREC Genomics track [8]. *Note: TREC is the largest academic forum for the evaluation of text retrieval systems*. Qualitatively, the relevance terms learned for each query appear highly relevant. It is especially interesting to note that many of the top *topic relevance *terms were not present in the query, were not identified as term variants by our normalization procedure, and were not identified as concept synonyms from external knowledge sources.

**Table 1:** Topic Relevance

**Query 200**	**Query 201**	**Query 202**
BILAG (0.6010)	B-RAF (0.54793)	1gangliosid (0.5424)
lupu (0.5650)	mutat (0.5039)	brain (0.5010)
anticardiolipin (0.3960)	RAF (0.4834)	gangliosid (0.4949)
immunodiffus (0.3870)	melanoma (0.4536)	accumul (0.4146)
isle (0.3750)	activ (0.4403)	abnorm (0.4008)
system (0.3331)	mutation (0.3661)	diseas (0.2690)
erythematosu (0.2954)	cell (0.3649)	asialo (0.2393)
antibodi (0.2820)	ERK (0.2508)	neuron (0.2323)
index (0.2776)	gene (0.2132)	protein (0.2067)
diseas (0.2488)	RAS (0.19943)	patient (0.1990)
activ (0.24514)	pathwai (0.1804)	cell (0.1985)
measur (0.2432)	human (0.1781)	lysosom (0.1895)
anticoagul (0.2320)	cancer (0.16233)	respons (0.1836)
clinic (0.2193)	autoinhibit (0.1617)	human (0.1793)
bacon (0.1807)	express (0.1570)	promin (0.1724)
patient (0.1551)	growth (0.1447)	mice (0.16800)
EM (0.1492)	phosphoryl (0.1183)	clinic (0.1673)
ISI (0.1425)	focus (0.1162)	gangliosidosi (0.1647)
hay (0.1404)	signal (0.1152)	phenotyp (0.1599)
score (0.1291)	tumor (0.1148)	storag (0.1559)
SLE (0.1196)	RAF1 (0.1128)	apoptosi (0.1326)

200: What serum [proteins] change expression in association with high disease activity in lupus?

201: What [mutations] in the Raf gene are associated with cancer?

202: What [drugs] are associated with lysosomal abnormalities in the nervous system?

Note: Terms are shown in stemmed form, acronyms have been capitalized, and the probabilities are not normalized.

### Concept model

Identifying biological concepts in text has traditionally relied on term matching techniques including the use of term normalization strategies, and the use of external knowledge sources for identifying synonymous terms. To capture latent relationships between concepts and terms, and to disambiguate concept instances, we incorporate sentence-level *concept-word *co-occurrence distributions. Grammatically, sentences express a complete thought and provide context for latent relationships between concepts and words. Such distributions are used to strengthen the relevance of concepts used within proper context, weaken the relevance of polysemous concept instances (concept terms identified in a context largely dissimilar to the concept-word distribution), and provide weight to potentially relevant sentences without explicitly matching a known concept term. Table 2 illustrates sample *concept-word *distributions from the 2007 TREC Genomics collection of full text articles. Potentially ambiguous acronyms like "*IG*" are disambiguated to "*Immunoglobulin G*" within the context of the concept *blood protein*. And conceptually related terms (hyponyms, hypernyms, and synonyms) such as *autoimmune disease *and *SLE *are associated within the concept *Lupus Erythematosus*.

**Table 2:** Concepts from 2007 TREC Genomics

**Concept 1: Blood protein**	**Concept 2: Lupus Erythematosus**	**Concept 8: Lysosome**
antibodi (0.4895)	lupu (0.6477)	lysosom (0.9999)
cell (0.2565)	SLE (0.4645)	cell (0.2326)
serum (0.2158)	system (0.3527)	protein (0.1848)
anti (0.2042)	patient (0.3482)	membran (0.1514)
plasma (0.1544)	erythematosu (0.3394)	endosom (0.1297)
protein (0.1498)	diseas (0.1749)	enzym (0.1019)
membran (0.0869)	antibodi (0.1172)	degrad (0.0912)
incub (0.0718)	cell(0.1109)	transport (0.0778)
human (0.0713)	anti (0.0779)	acid (0.0704)
monoclon (0.0677)	nephriti (0.0744)	compart (0.0644)
express (0.0623)	mice (0.0618)	storag (0.0597)
bind (0.0596)	autoantibodi(.06)	target (0.0582)
concentr (0.0541)	clinic (0.0579)	pathwai (0.0540)
beta (0.0513)	autoimmun (0.0557)	accumul (0.0536)
IG (0.0507)	DNA (0.0545)	diseas (0.0528)
alpha (0.0477)	human (0.0479)	human (0.0522)
mous (0.0477)	syndrom (0.0429)	express (0.0474)
rabbit (0.0459)	factor (0.0428)	cathepsin (0.0456)
CD (0.0434)	ISI (0.0424)	organel (0.0414)
glycoprotein (0.0423)	IG (0.0382)	golgi (0.0402)

Notes: Each concept is defined by the probability of a sentence containing the concept generating the term.

Concept names, e.g., "Blood Protein" for Topic 1, are extracted from the UMLS Metathesaurus.

Terms, sans acronym, are shown in stemmed form.

The passage *concept *model is summarized as follows:

1. Concept term instances are identified during query processing (refer to the query processing section).

2. The likelihood of each sentence (within a candidate passage) being generated for a given concept is determined from the concept-word co-occurrence distribution. The distribution is generated for all words co-occurring with an instance of a concept term in the same sentence. The likelihood of each sentence *k *within a candidate passage of generating a given query concept c_*j *_is estimated using equation (9).

(9) $$p_{d}(c_{j}|s_{k})\approx \sum_{i=1}^{\left|S\right|}p(w_{i}|c_{j})p(c_{j})$$

3. As shown in equation (10), the probability of a concept being generated for a given sentence is determined using Jelinek-Mercer style linear-weighting of the Boolean presence of the concept weighted by the likelihood of the concept term instance distinctly representing the concept, and the likelihood of the sentence given the concept (equation 9).

(10) *p*(*c*_*j *_| *s*_*k*_) = λ *(*present*(*c*_*j*_)* Γ) + (1 - *λ*)* *p*_*d*_(*c*_*j*_|*s*_*k*_)

*λ *is the linear weighting between concept presence and the likelihood of the concept from the *concept-word *distribution. We set *λ *= 0.8 to emphasize the presence of resolved concept terms. Γ represents the likelihood of a concept-term instance distinctly representing concept *c*_*j*_, which we approximate with the normalized *IDF (0–1) *of the concept term.

4. Finally, the probability of passage *P *generating query *Q *for concept model *θ*_*c *_is shown in equation (11).

(11) $$p(Q|P,\theta_{c})=\sum_{k}^{\left|S\right|}\sum_{j}^{\left|C\right|}\log(1+p(c_{j}|s_{k})$$

*C *is the set of query concepts, and *S *is the minimum set of contiguous sentences covering the maximum number of distinct query concepts.

### Term model

Sentence-level *term *co-occurrence distributions are used with term matching within the *term *model. The *term *model uses the same formulation as the *concept *model and is based on the likelihood of passage terms co-occurring.

### Document model

The Jelinek-Mercer language model is used to capture document context (12). We set *λ *= 0.8.

(12) *p*(*q *| *d*_*i*_) = Σ_*wq*_log(λ *P_*ml*_(*w*|*d*) + (1 - *λ*)* *p*(*w*_*k*_|*C*))

*P*_*ml*_(*w*|*d*) = *tf*_*d*_/*doclen *represents the likelihood of a term given a document, *P*(*w*|*C*) represents the term collection frequency.

### Dimensional indexing model

**Figure 2:**
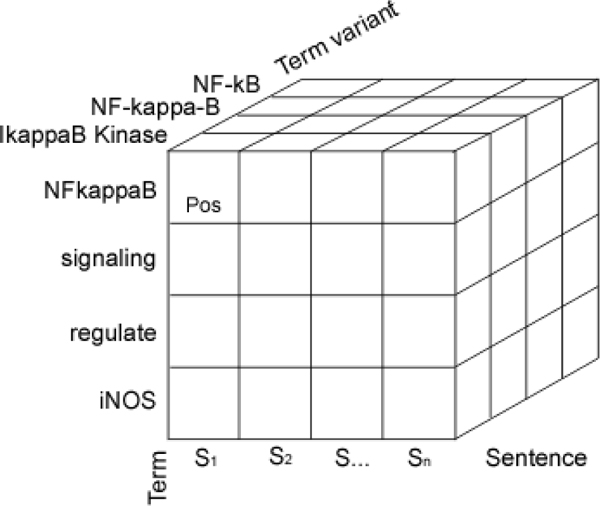
**Dimensional term index (paragraph)**.

We use a dimensional indexing model to efficiently aggregate term co-occurrence statistics. The *grain *of the *index *is an individual *term variant*. Figure 2 illustrates a cube representing a paragraph within the *term index*. For simplicity, the document dimension is not shown. Each document consists of a sequence of paragraphs, each paragraph consists of a sequence of sentences, each sentence consists of a sequence of terms, and each term consists of one or more term variants.

By indexing each individual word, queries can be developed for searching single- and multi-word terms. In the data warehousing literature, this model is refered to as a *star schema *[9,10]. A more detailed treatment of the dimensional indexing model can be found in Urbain, Goharian, and Frieder [11].

The indexing process includes:

1. *Lexical Partitioning*: Documents are parsed into paragraphs, and sentences.

2. *Tokenization*: Acronyms and their long-forms are identified using the [12]. Sentence terms are tokenized, stop words removed, and lexical variants are generated [13].

3. *Indexing*: Each term along with its long-form expansion and lexical variants are stored in the index with the same positional information.

### Query processing

Structured query generation for concept identification is illustrated with the following query: "*Provide information about the role of the gene PRNP (prion protein) in the disease Mad Cow Disease*".

1. Sentences are extracted, and acronyms and their long-forms are identified: PRNP (PRioN Protein).

2. Part-of-speed tagging is performed using our 2^nd ^order statistical Hidden Markov Model tagger: ... *role_NN of_II the_DD gene_NN PRNP_NN (_(prion_NN protein_NN)_) in_II the_DD disease_NN Mad_NN Cow_NN Disease_NN*.

3. Stop and function words are removed.

4. Candidate concepts are identified by locating non-recursive noun phrases ("noun chunks"): [gene PRNP], [prion protein], [Mad_NN Cow_NN Disease_NN].

5. Candidate concepts are verified in the index, and resolved using the UMLS Metathesaurus^®^, and Entrez Gene databases [14]. If an entity is successfully resolved, all synonyms and one level of hyponyms (from the UMLS) are included.

6. If the synonym is considered ambiguous, it is not included. We consider a term ambiguous if either:

1) The synonym's normalized IDF (NIDF) is < 0.1. (IDF = log (N/df) normalized to between 0 and 1).

2) The synonym correlates with the long-form in less than 20% of all instances within the acronym table.

Resolved concepts and synonyms are shown in Table 3.

**Table 3:** Entity resolution

**Resolved concepts**	**Synonyms**
[Encephalopathy, Bovine Spongiform]	[Mad Cow Disease]
	[MCD]
	[BSE]
	[Creutzfeldt-Jakob disease]
	[CJD]
[PRNP gene]	[prion protein]
	[prnp]

Next, using the top 1000 paragraphs retrieved using the *document *retrieval function (12), we perform concept search:

1. The position of all term variants of each concept is retrieved from the dimensional index by paragraph.

2. A minimum-spanning tree is constructed from the adjacency list by determining the maximum number of distinct concepts identified within the shortest lexical distance.

3. Finally, the passage boundary based on the first and last occurrences of distinct concepts is expanded out to include sentence boundaries.

Passage level concept search is illustrated with the following query: *"Exact reactions that take place when you do glutathione S-transferase (GST) cleavage during affinity chromatography"*.

First, concepts and term variants are identified:

*Cleavage: *[cleavag], [merogenesi], [cytokinesi]]

*Affinity purification*: [affin, purif], [affin, chromatographi]]

*Glutathione S-transferase*: [glutathion, s, transferase], [gst]]

Second, the index is searched for all concept term variants.

Third, passages are identified: "***affinity chromatography***, *and purified Mce1A and Mce1E, free of the fusion partner, were recovered following specific proteolytic ****cleavage ****of the ****GST***"

Fourth, passages are expanded to sentence boundaries: *"The fusion proteins were purified to near homogeneity by ****affinity chromatography***, *and purified Mce1A and Mce1E, free of the fusion partner, were recovered following specific proteolytic ****cleavage ****of the ****GST ****portion by thrombin protease*."

## Results

Results on the 2007 TREC Genomics track of *162,000 *full-text documents (~10 GB), and 36 official topic queries are listed in Table 4[8]. All results are for *automatic retrieval *(no user intervention). Included in our evaluation are comparisons to the top automatic runs in each category from the 2007 TREC Genomics track (reference Table 4). Statistical significance measurements were made using the paired Wilcoxon signed-rank test. As an additional baseline, we've included our top 2007 TREC Genomics track submission [13] in Table 5. This model used the same preprocessing, but required explicit concept term matching without the benefit of distributional evidence for disambiguation and context. Evidence of concepts and query terms was combined using a linear weighting scheme. This technique worked reasonably well (2^nd ^place) for the original passage overlap measurement, but significantly underperforms the proposed model for all measurements.

**Table 4:** Results 2007 TREC Genomics collection (MAP)

**Model**	**Doc**	**Passage**	**Passage2**	**Aspect**
Top TREC*	0.3105	0.0976	0.1097	0.2494
Median TREC	0.1954	0.0565	0.0391	0.1272
TREC 2007 Submission	0.2385	0.09742	0.1647	0.05164
Document model	0.2363	-	-	-
Topic model No relevance	0.2034	-	-	-
Topic-relevance model	0.2605	0.0898	0.0452	0.1383
Concept model	0.3381	0.1087	0.0579	0.1907
Term model	0.3226	0.1053	0.0557	0.1856
Concept+Term models	0.3443	0.1100	0.0588	0.2145
Doc+Concept +Term+Topic-relevance *Min Spanning Passage*^5^	0.3554 (+14.46%) *p = 0.0582*	**0.1214 (+24.39%) *p = 0.0321***^†^	0.0681 (-37.92%)	0.2412 (-3.29%)
Doc+Concept +Term+ Topic-relevance *Max Spanning Passage*^6^	**0.3576 (+15.17%) *p = 0.0504***	0.1093 (+11.99%)	**0.1280 (+16.68%) *p = 0.0834***	**0.2596 (+4.08%)**

†Statiscally significant using Wilcoxon signed rank test (p < 0.05).

**Table 5:** Top Results for TREC 2007 Genomics Track

***Category***	***Run***	***Contributor***
*Document*	*NLMFusion*	*Demner-Fushman, et al*., [15]
*Passage*	*UICGenRun2*	*Zhou, Yu*, [16]
*Passage*	*NLMFusion*	*Demner-Fushman, et al*., [15]

To get a better understanding of the effectiveness of our proposed *topic relevance *model, we include the results from automatically learned topic models from our earlier work [4]. These topic models lack a model of relevance with respect to the user's information need. The *topic relevance *model outperforms passages scored using the general *topic model *by *28.07%*. This is clearly a more effective technique for incorporating topic models in information retrieval.

To understand the contributions of each component model, we have listed the results for ranking passages by each model individually along with the results of the full passage retrieval model (integrating evidence from the *document, concept*, *term, and topic relevance *models). The percentage improvements shown for the full passage retrieval model are relative to the top results in each category from all submissions to the 2007 TREC Genomics track.

The use of the minimal spanning distance of distinct concepts expanded to sentence boundaries for defining passages for evaluation resulted in a significant improvement for the *Passage *measurement which emphasizes precision, but less effective results for the *Passage2 *and *Aspect *measurements which place greater emphasis on recall. As an alternative, we evaluated submitting passages by the maximal spanning distance of all concepts (non-distinct). This resulted in significant improvements in the *Passage2 *and *Aspect *scores, no significant difference in the *Document *score, and a modest decrease in the *Passage score*. The overall results show significant improvements in *Document *and *Passage *retrieval using *min spanning distance passages*, and also improvements for *Passage2 *retrieval using the *max spanning distance passages*.

## Discussion and related work

The proposed passage retrieval model exceeded the top results in each category, and demonstrated statistically significant improvements in document and the original passage retrieval measurement across a large test collection of genomics literature. The model can be used to help disambiguate polysemous terms and provide weight to potentially relevant passages without explicit term matching by capturing term co-occurrence distributions within context, and incorporating these distributions within a statistical relevance model.

Combining evidence from all component models rather than using evidence from any individual component alone achieved the best results. Examination of relevant passages returned from the system indicated that the use of term distributions within the *concept*, *term, and topic relevance *models had the most significant impact on passages where the system was able to identify only one or a small number of potentially ambiguous query concepts or terms respectfully. In these cases, the distributions were helpful in disambiguating acronyms and terms for biological concepts. For example, in the 2005 TREC query: *How to "open up" cell through "electroporation," we were able to identify only one distinct concept: electroporation, as the acronym EPT*.

The system was able to disambiguate the use of the acronym *EPT *in the following two passages and rank the second passage significantly higher even though *electroporation *is often used to treat endocrine pancreatic tumors.

Passage 1: ...malignant potential among endocrine pancreatic tumors (**EPTs**) varies greatly and can frequently not be predicted using histopathological parameters...

Passage 2: ... **EPT**, which uses pulsed electric fields in combination with a chemotherapeutic agent is being developed to treat human pancreatic tumors...

In the 2007 TREC query: *What serum [proteins] change in association with high disease activity in lupus?*

The system was able to only identify the concept *lupus *in the form of *SLE *(Systemic Lupus Erythematosus) in the following passage. We were not able to identify *serum *in the form of *sera*, but we were able to accurately identify this passage as relevant, and rank it higher than less relevant passages about lupus, which did not deal with blood serum.

Passage: ... *SLE sera were used. The most marked nuclear staining occurred with sera from patients with active disease*...

In passages where multiple distinct concepts are identified, the distributions had only a marginal effect in the ranking as enough evidence is provided by the presence of other query concepts for accurate disambiguation.

The *topic relevance *model clearly improved results, exceeding the automatically learned topic models by *28.07%*, and our 2007 TREC results, which had the benefit of using concepts. The *topic-relevance *model also exceeded the median results of the track in all categories, and improved the performance of our composite passage retrieval model. Most importantly, the *topic relevance *model significantly outperforms general topic modeling using only a fraction of the computational resources.

To the best of our knowledge, there has not been prior work that models topics, concepts, and terms with distributional evidence within the framework of an undirected graphical model. Blei, Jordan, and Ng [17] introduced the idea of using hierarchical Bayesian models for applications in information retrieval including the estimation of latent Dirichlet hyperparameters using variational Bayes inference. They reported empirical results only and did not analyze precision with respect to user queries. Liu and Croft [18] introduced a cluster model for document retrieval; and Azzopardi, Girolami, and van Rijsbergen [5], and Wei and Croft [6] used an LDA-based language model for document retrieval. Both techniques demonstrated good results, but did not exceed the results of top-performing relevance-based language models [7].

Using word co-occurrence information has a long history in word sense disambiguation research and goes back to the famous dictum by J. R. Firth: "*you shall know a word by the company it keeps*" [19]. Yarowsky [20] showed that with high probabiity a polysemous word has one sense per discourse.

Several researchers have made contributions to modeling term dependencies. Most work has focused on phrases, term proximity, and co-occurrence for pairs of terms [21,22]. Metzler and Croft [23] developed a Markov Random field for modeling single terms, ordered phrases, and unordered phrases. They explored a number of independence assumptions and optimized their model for mean-average precision rather than likelihood to achieve their best results.

Using retrieval of fixed length passages of text to improve retrieval of relevant documents is based on the premise that only a small portion of each relevant document is relevant to a user's query. Similarity coefficients are computed at the passage level, and the highest scoring passage or some combination of the scores of individual passages is used to compute a document's similarity coefficient [24-26]. Callan [27] used a combination score with document and passage level evidence to obtain their best results. These efforts focused on fixed length passages of text and did not include multiple levels of document context and semantic evidence. Tellex [28] performed a quantitative evaluation of passage retrieval algorithms used by question-answering systems. Common to all three top performing algorithms is a non-linear boost to query terms that occur very close together in a candidate passage.

## Conclusion

We presented a passage relevance model based on an undirected graphical model *(Markov Random Field)*, and methods for modeling concepts, terms, and topic relevance as potential functions within the model. Using relevance modeling, we've introduced a new, more effective method for incorporating topic modeling into information retrieval applications that is also computationally efficient. Topic modeling using relevance outperformed automatically generated topic models by *28.07%*.

The full model outperforms models of query terms, concepts, document, or passage relevance alone. Modeling query topic relevance improves the overall performance of the model and significantly outperforms topic models without relevance modeling. The model exceeds the top results in each category of retrieval as assessed by the 2007 TREC Genomics track and the results are *statistically significant *for automatic document and passage retrieval.

## Competing interests

The authors declare that they have no competing interests.

## Authors' contributions

Jay Urbain completed this research as a PhD student under the supervision of Ophir Frieder and Nazli Goharian.
